# *PTGDR* gene expression and response to dexamethasone treatment in an in vitro model

**DOI:** 10.1371/journal.pone.0186957

**Published:** 2017-10-31

**Authors:** Elena Marcos-Vadillo, Asunción García-Sánchez, Catalina Sanz, Ignacio Davila, María Isidoro-García

**Affiliations:** 1 Institute for Biomedical Research, IBSAL, Salamanca, Spain; 2 Department of Biomedical and Diagnostic Sciences, University of Salamanca, Salamanca, Spain; 3 Department of Microbiology and Genetics, University of Salamanca, Salamanca, Spain; 4 Department of Allergy, University Hospital of Salamanca, Salamanca, Spain; 5 Department of Clinical Biochemistry, University Hospital of Salamanca, Salamanca, Spain; 6 Department of Medicine, University of Salamanca, Salamanca, Spain; Wayne State University, UNITED STATES

## Abstract

Asthma is a multifactorial pathology influenced by environmental and genetic factors. Glucocorticoid treatment decreases symptoms by regulating genes involved in the inflammatory process through binding to specific DNA sequences. Polymorphisms located in the promoter region of the Prostaglandin D Receptor (*PTGDR*) gene have been related to asthma. We aimed to analyze the effect of *PTGDR* promoter haplotypes on gene expression and response to corticosteroid therapy. A549 lung epithelial cells were transfected with vectors carrying four different *PTGDR* haplotypes (CTCT, CCCC, CCCT and TCCT), and treated with dexamethasone. Different approaches to study the promoter activity (Dual Luciferase Reporter System), gene expression levels (qPCR) and cytokine secretion (Multiplexed Bead-based Flow Cytometric) were used. In addition, *in silico* analysis was also performed. Cells carrying the TCCT haplotype showed the lowest promoter activity (p-value<0.05) and mRNA expression levels in basal conditions. After dexamethasone treatment, cells carrying the wild-type variant CTCT showed the highest response, and those carrying the TCCT variant the lowest (p-value<0.05) in luciferase assays. Different transcription factor binding patterns were identified *in silico*. Moreover, differences in cytokine secretion were also found among different promoter haplotypes. Polymorphisms of *PTGDR* gene influence basal promoter activity and gene expression, as well as the cytokine secretory pattern. Furthermore, an association between these positions and response to corticoid treatment was observed.

## Introduction

In the last decades, there has been a great increase in prevalence rate of allergic respiratory diseases, being considered a priority line in biomedical research. Corticosteroids constitutes the mainstay treatment for patients with allergic diseases and asthma [1–3]. In asthma, glucocorticoid (GC) treatment reduces symptoms, recovers pulmonary function, and decreases bronchial hyperresponsiveness and exacerbations [4]. GCs exert their action through the specific receptor GR, (Glucocorticoid Receptor). The GC·GR complex is transported into the cellular nucleus, regulating the expression of many genes involved in the inflammatory response. These actions are mediated by specific binding sites carrying consensus sequences (GRE, glucocorticoid response element) in the target genes [5–7]. Many of the anti-inflammatory effects of the corticosteroid therapy are attributed to direct inhibition of transcription factors AP-1 (Activator Protein-1) and NF-κB (Nuclear Factor kappa B), regulating the levels of different molecules involved in the inflammatory process (IL-1β, TNF-α, IL-2, IFN-γ, IL-6, IL-12, RANTES) [8,9]. Furthermore, glucocorticoids suppress Th2 cells and cytokine production by inhibiting the transcription factor GATA3, critical for the expression of IL-4, IL-5 and IL-13 [10], or by inhibiting the MAPK pathway. It has been estimated that 5–10% of asthmatics do not respond well to corticosteroids, developing severe asthma [11]. According to some studies of gene expression profiles, there is a genetic predisposition to glucocorticoid resistance and severe asthma [12,13].

Asthma is a multifactorial pathology of complex aetiology including genetic and environmental factors [14]. Different linkage and association studies have identified numerous genetic markers linked to allergic disease on chromosome 14 [15,16], which include the *PTGDR* gene. It encodes a transmembrane receptor of prostaglandin D_2_ (PGD_2_), being PGD_2_ the major metabolite of arachidonic acid produced by activated mast cells during an allergic reaction [17]. PTGDR is expressed in immune cells, but also in platelets, cells of the central nervous system, ciliated and non-ciliated bronchial cells and alveolar epithelial type I and type II cells [18]. In recent years, certain polymorphisms and specific haplotypes and diplotypes of the promoter region of *PTGDR* have been associated to allergy and asthma [19–21]. In addition, epigenetic aspects of the *PTGDR* promoter have also been associated with asthma [22]. All these factors would affect the capacity of *PTGDR* promoter regions to bind transcription factors, modifying their expression levels and increasing susceptibility to the disease.

Considering all these data, *PTGDR* has been considered as a potential therapeutic target in the asthmatic disease investigation. Our purpose was to study how the polymorphic combinations on *PTGDR* promoter could affect its activity, and evaluate its relationship with the responsiveness to corticoid therapy.

## Methods

### Cell culture

A549 cells (human adenocarcinoma alveolar basal epithelial cells, type II) were kindly provided by Dr Odero (CIMA, University of Navarra) and maintained in RPMI with 10% Fetal Bovine Serum (FBS) (Gibco, Invitrogen-Life Technologies, MA, USA) at 37°C in a humidified atmosphere with 5% CO_2_. To perform luciferase and expression experiments, cells were grown in RPMI supplemented with 10% Charcoal Stripped FBS (Gibco, Invitrogen-Life Technologies, MA, USA) to avoid possible interference with serum steroids [23,24].

### Exposure to corticoid

A time-course test was performed with different dexamethasone (DEX) concentrations to analyse cell culture response. Cell culture response was monitored by *CYP3A5* expression analysis [25]. Two DEX concentrations (2.5 x 10^−6^ M and 2.5 x 10^−7^ M) and four treatment times (12 h, 24 h, 36 h and 48 h) were tested. A final DEX concentration of 2.5 x 10^−6^ M, and two experimental times, 12 h and 36 h, were chosen.

### Identification of *PTGDR* promoter variants

Genomic DNA from patients and from A549 cells was isolated with the MagNA Pure Nucleic Acid Isolation Kit using the MagNA Pure Compact instrument (Roche Applied Science, IN, USA), and amplified by polymerase chain reaction (PCR) using the oligonucleotide sense primer 5’-CTCAGTTTCCTCGCCTATGC-3’ and the anti-sense primer 5’-GAGTGAAGGCTGCGGAAGGG-3’. PCR products were cleaned with exoSAP-IT (USB-Affimetrix, OH, USA) prior to sequencing in an ABI Prism 3100 Genetic Analyzer (Applied Biosystems-Thermo Scientific, MA, USA).

### Plasmid construction

*PTGDR* promoter constructs of 653 bp were generated by PCR amplification of genomic DNA from asthmatic patients. The most frequent combinations at positions -613, -549, -441 and -197, differently expressed in patients were selected: CTCT (wild-type) (haplotype frequency in controls 0.26 vs 0.23 in patients), CCCC (0.11 vs 0.12), CCCT (0.31 vs 0.32) and TCCT (0.08 vs 0.10). Primers used incorporated recognitions sites for *XhoI and BglII*. All constructs were sequenced to exclude additional mutations.

For promoter activity assays, the above-described constructs were ligated and cloned into multicloning sites of the firefly luciferase pGL3-Basic vector (Promega, WI, USA). For expression assays, the above-described constructs were ligated and cloned into the RC208087 (OriGene, MD, USA) vector containing the *PTGDR* coding sequence. Plasmid DNA was purified with a Maxiprep kit (Qiagen, Germany). All plasmids were verified by sequencing.

### Transient transfection and treatment

Cells were seeded in antibiotic free medium until cell confluence reached 50–70%, and then transfected using Lipofectamine Reagent 2000 (Invitrogen, Thermo Fisher Scientific, MA, USA) according to the manufacturer’s protocol. After 5 hours, transfection medium was removed and replaced with fresh RPMI with 10% charcoal stripped serum, antibiotics and dexamethasone (DEX) or vehicle (ethanol). A condition of no-treatment was studied as a basal condition. DEX (Sigma-Aldrich, Germany) was dissolved in absolute ethanol according to manufacturer's instructions.

### Luciferase assays

A549 cells were seeded at density of 5x10^4^ cells/well in 24-well plates, and co-transfected with 500 ng of the firefly luciferase reporter plasmid described above (pCTCT-*luc*, pCCCC-*luc*, pCCCT-*luc*, and pTCCT-*luc*), and 10 ng renilla luciferase reporter plasmid pRL-SV40 vector (Promega, WI, USA) as internal control. Cells were collected and lysed after 12 h and 36 h of DEX or ethanol treatment for luciferase activity measurement. Analysis was performed in a FLUOstar OPTIMA Luminometer (BMG LabTech, Germany) using the Dual-Luciferase Reporter Assay System (Promega, WI, USA). Data were represented as relative firefly luciferase normalized to renilla luciferase activity as Relative Luciferase Units (RLU). Each assay condition was performed using 3 replicates and the global experiment was replicated 3 times. In the evaluation of the treatment with corticoids, the values obtained with the vehicle (ethanol) were considered the baseline situation, so they were subtracted from the values obtained with DEX.

### Expression assays

Cells were seeded into 6-well plates and transfected with 1 μg of expression plasmid containing the *PTGDR* promoter variants and the *PTGDR* coding sequence described above (pCTCT-*PTGDR*, pCCCC-*PTGDR*, pCCCT-*PTGDR* and pTCCT-*PTGDR*). Total amounts of DNA (2.5 μg) per well were balanced by adding pUC18 plasmid. Cells were collected after 12 h and 36 h of DEX or ethanol treatment, and total RNA was isolated using the RNeasy Plus Mini kit (Qiagen, Germany) according to manufacturer’s instructions. DNAse treatment was performed using Turbo DNAse (Ambion, Thermo Fisher Scientific, MA, USA). cDNA was generated using SuperscriptTM III (Invitrogen, Thermo Fisher Scientific, MA, USA). Relative quantitative PCR was performed using the LightCycler 480 Instrument and SYBR Green I Master (Roche Applied Science, IN, USA). MIQE guidelines were followed in the gene expression study. According to these, two genes (GAPDH and TPB) were initially selected as potential candidates. A correlation study was developed in A549 cells in both conditions (with and without dexamethasone treatment). High stability was observed for both genes (r = 0.992, p = 0.001) and in both condition (r = 0.999, p<0.001). Considering these results, *GAPDH* was selected based, as well, on its broad use in previous studies developed in the same cell line treated with corticoid [26–29]. The following primers were used: *PTGDR* forward primer 5’-GGCATGAGGCCTAAAAATGAG-3’ and reverse primer 5’-CCTTGACATCCTTAAATGCTCC-3’; *GAPDH* forward primer 5’-CTCTGCTCCTCCTGTTCGAC-3’ and reverse primer 5’-ACGACCAAATCCGTTGACTC-3’. Fold induction was calculated using the formula 2^-(ΔΔCt)^ [30]. PCR product specificity was monitored using post-PCR melt curve analysis. Data were expressed as fold change relative to RNA levels for control cells (transfected cells with control vector pCMV-XL5) in the same conditions. The experiments were performed in duplicate. In the evaluation of the treatment with corticoids, the values obtained with the vehicle (ethanol) were considered the baseline situation, so they were subtracted from the values obtained with DEX.

### *In silico* characterization of transcription factors

Two bioinformatics algorithms were used to identify differences of putative biding sites for transcription factors based on polymorphic changes -613C>T, -549T>C, -441C>T and -197T>C in *PTGDR* promoter: MatInsprector software (Genomatix Software GmbH) (www.genomatix.de) and Transfac (BioBase) (www.gene-regulation.com/). The analysis included the *PTGDR* promoter region used in the plasmid construction.

### Multiplexed bead-based flow cytometric assay

Cytokine analyses were undertaken on culture medium supernatants of the cells transfected with each expression vector (pCTCT-*PTGDR*, pCCCC-*PTGDR*, pCCCT-*PTGDR* and pTCCT-*PTGDR*). Cell culture supernatants were centrifuged before to be stored at -80°C, and allowed to cool to room temperature before bead-based cytometric assays were set up. For the cytokine analysis, we used the Bio-Plex Pro™ Human Cytokine standard 27-plex, Group I kit (Bio-Rad, CA, USA) following the manufacturer's instructions. This multiplex assay allowed detecting IL-1β, IL-1ra, IL-2, IL-4, IL-5, IL-6, IL-7, IL-8, IL-9, IL-10, IL-12(p70), IL-13, IL-15, IL-17, Eotaxin, FGF-basic, G-CSF, GM-CSF, IFN-γ, IP-10, MCP-1, MIP-1α, MIP-1β, PDGF-BB, RANTES, TNF-α and VEGF. Cytokine standards, controls and samples were added (50 μL total volumes) to 96 well plates in duplicates and incubated with beads conjugated with specific antibodies. After washing, biotinylated detection antibodies were incubated with the bound cytokines. Three washes to remove antibodies excess were performed, and the streptavidin reagent was added. A final wash was followed by resuspension in Assay Buffer for analysis using the Bio-Plex ManagerTM 5.0 software (Bio-Rad, CA, USA). An eight-point standard curve was constructed and the concentration of each cytokine calculated against this curve through a logistic regression model. All samples were measured in duplicate.

### Statistical analysis

All data were presented as mean ± SD. Statistical analysis was performed using SPSS 21.0 (IBM, IL, USA). Measures of central tendency, measures of dispersion, Kolmogorov-Smirnov, Levene, Kruskal-Wallis, ANOVA and Wilcoxon test were calculated. P < 0.05 was considered significant.

## Results

### Different genetic variants of *PTGDR* promoter show different promoter activity

**Fig 1:**
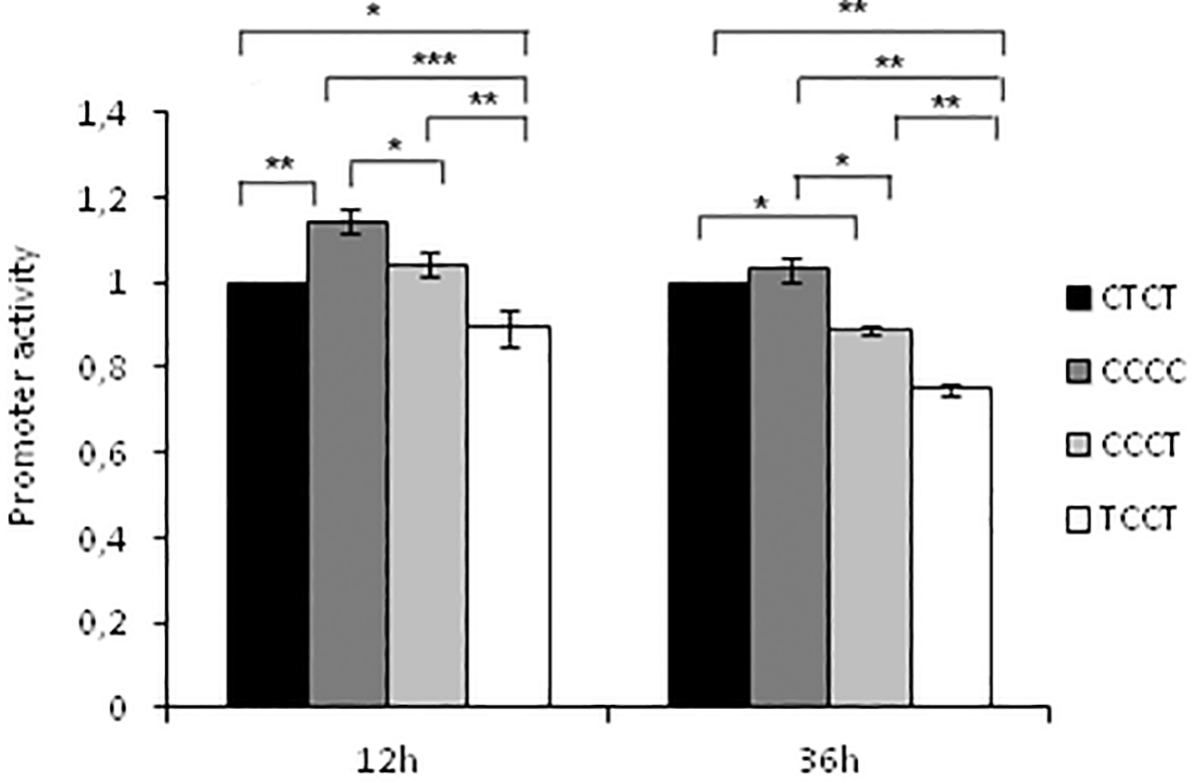
*PTGDR* polymorphic variants modify its basal promoter activity. A549 cells transfected with reporter plasmid bearing the CTCT (wt) or CCCC, CCCT, TCCT (mutated) *PTGDR* promoter sequences. Kolmogorov-Smirnov, ANOVA and Bonferroni test were performed. Data are represented as mean ± SD of Relative Luciferase Units (RLU) (*Fisher-p<0.05; **Fisher-p<0.01, ***Fisher-p<0.001).

We analysed the basal promoter activity of *PTGDR* for common haplotype variants in asthmatic patients: CTCT, CCCC, CCCT and TCCT (positions -613, -549, -441 and -197). Significant differences in promoter activity were detected between haplotypic combinations after luciferase reporter plasmid analysis in the A549 cell line (Fig 1).

Promoter activity after normalization was expressed as relative luciferase units (RLU), with a value of 1 assigned to the wild-type variant CTCT. After 12 h, mean RLU values were 1.14±0.03 for CCCC, 1.04±0.03 for CCCT and 0.89±0.04 for TCCT. Cells carrying both mutant alleles -613T and -549C (haplotype TCCT) showed the lowest reporter activity at 12 h (vs. CTCT p-value = 0.013; vs. CCCC p-value<0.001; vs. CCCT p-value = 0.002). In contrast, the CCCC haplotype showed the highest reporter activity (vs. CTCT p-value = 0.002; vs. CCCT p-value = 0.019; vs. TCCT p-value <0.001). After 36 h, mean RLU values were 1.03±0.03 for CCCC, 0.89±0.01 for CCCT and 0.075±0.01 for TCCT. Once more, it was confirmed that the variant carrying the TCCT haplotype remained with the lowest expression (vs. CTCT p-value = 0.005; vs. CCCC p-value = 0.003; vs. CCCT p-value = 0.001). The CCCT haplotype showed also a lower expression than wild-type CTCT (p-value = 0.025) and CCCC (p-value = 0.028) haplotypes.

### DEX treatment increases promoter activity with a haplotype-dependent response

**Fig 2:**
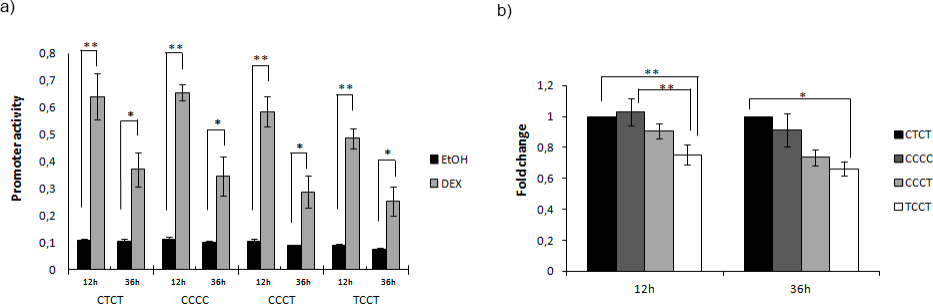
Dexamethasone treatment modifies *PTGDR* promoter activity. A549 cells transfected with reporter plasmid bearing the CTCT (wt) or CCCC, CCCT, TCCT (mutated) *PTGDR* promoter sequences and stimulated with 2.5x10^-6^ M DEX or ethanol (vehicle) for 12 or 36 h. a) Each value represents the mean ± SD of Relative Luciferase Units (RLU) obtained in each condition. b) Data represented as mean ± SD of RLU, after vehicle effect removal normalized to pCTCT-*luc* vector values. Kolmogorov-Smirnov, ANOVA and Bonferroni test were performed. (*Fisher-p<0.05; **Fisher-p<0.01).

A549 cells were split into two groups: the first group was treated with 2.5x10^-6^M DEX, and the second group was treated with an equivalent dose of ethanol (DEX vehicle) as baseline. We analysed the influence of treatment with DEX on the *PTGDR* promoter activity at 12 and 36 hours (Fig 2A). Our results showed that A549 treated cells had a significantly higher promoter activity than untreated cells. This luciferase signal increase was more evident after 12 hours of treatment, although statistical significance was maintained both at 12 and 36 hours (p-value = 0.002 and p-value = 0.024 respectively).

Cells carrying the mutant variants at -613 and -549 positions (TCCT) maintained the lowest levels of promoter activity in response to corticoid. After 12 hours of 2.5x10^-6^M DEX treatment, this haplotypic variant showed a 0.75±0.06-fold in luciferase signal. This difference reached statistical significance versus CTCT (value 1; p-value = 0.006) and CCCC (1.02±0.089-fold; p-value = 0.003) variants. After 36 hours of exposure to dexamethasone, the wild-type CTCT showed the highest promoter activity, whereas the TCCT variant remained as the lowest active haplotype, with a value of 0.66±0.05-fold (p-value = 0.036). At this experimental time, A549 cells carrying CCCC and CCCT haplotypes did not show significant differences (Fig 2B).

### Effect of *PTGDR* promoter polymorphisms on gene expression

Once promoter activity differences were established, we proceeded to the gene expression analysis. The four promoter haplotypes, CTCT, CCCC, CCCT and TCCT, were cloned into expression vectors carrying the complete coding sequence of *PTGDR* gene.

**Fig 3:**
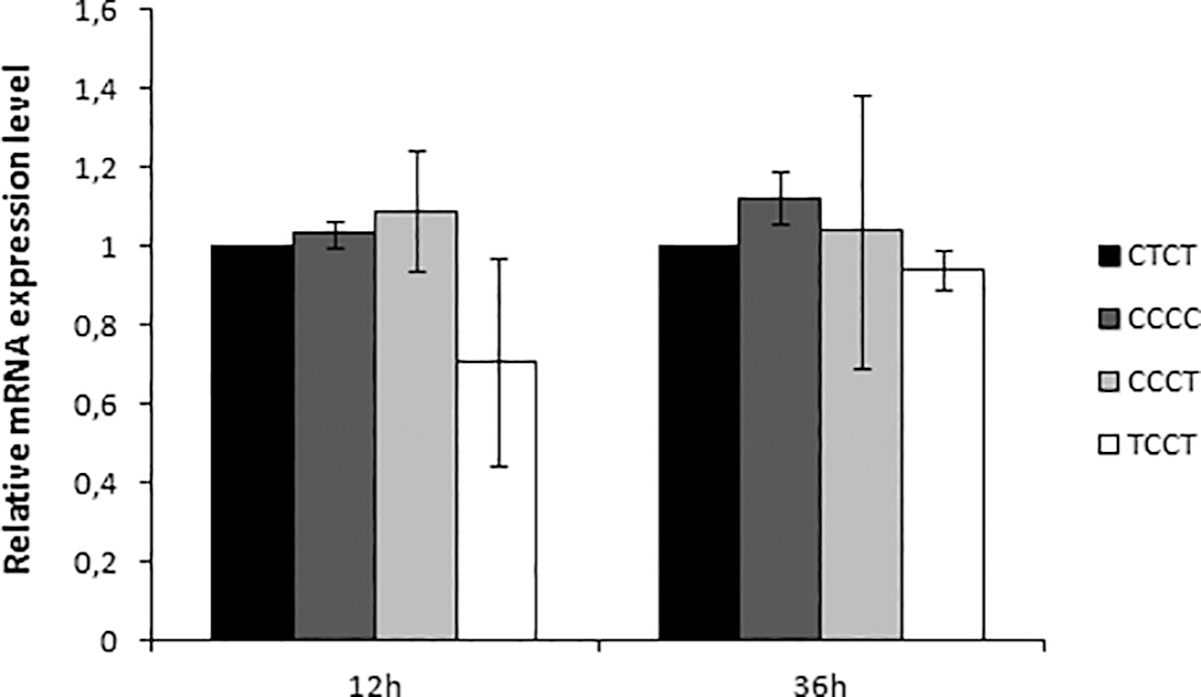
Quantitative real-time PCR analysis of *PTGDR* expression in no-treatment condition. A549 cells were transfected with expression plasmids of 653 bp bearing the CTCT (wt) or CCCC, CCCT, TCCT (mutated) *PTGDR* promoter sequences, followed by the gene coding sequence. Changes in *PTGDR* expression were related to *GAPDH* as a housekeeping gene. Data were represented as mean ± SD, and shown as fold increase relative to mRNA levels for cells with CTCT (wt) haplotype (value 1).

Considering the wild-type variant CTCT as value 1, at 12 hours of culture the lowest expression levels were detected for the TCCT haplotype (0.71±0.27-fold in TCCT, vs. 1.03±0.03-fold in CCCC and 1.09±0.15-fold in CCCT). A similar situation was observed after 36 hours although these differences between variants did not reach statistical significance (Fig 3). Kolmogorov and Kruskal-Wallis test were performed.

### DEX treatment modifies *PTGDR* expression levels

**Fig 4:**
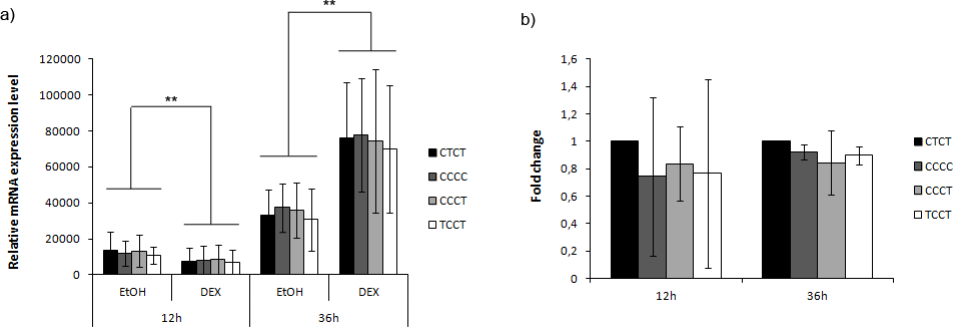
Dexamethasone treatment modifies *PTGDR* expression levels. A549 cells were transfected with expression plasmid bearing the CTCT (wt) or CCCC, CCCT, TCCT (mutated) *PTGDR* promoter sequences followed by the gene coding sequence, and stimulated with 2.5x10^-6^ M DEX or ethanol (vehicle) for 12 or 36 h. Values obtained with ethanol were considered the baseline situation. Kolmogorov, Wilcoxon and Kruskal-Wallis test were performed. a) Each value represents the mean ± SD of mRNA expression levels obtained in each condition. b) Data represented as mean ± SD, after vehicle effect removal, and shown as fold increase relative to mRNA levels for cells with CTCT (wt) haplotype (value 1). (**Wilcoxon-p <0.01).

Cells treated with 2.5x10^-6^M DEX followed a time dependent pattern in *PTGDR* mRNA expression levels. After 12 hours of treatment, *PTGDR* expression underwent an important decrease compared to controls with ethanol (p-value = 0.007) (Fig 4A). This decrease was detected in all variants. In contrast, when we extended the experimental time of corticoid exposure up to 36 hours, *PTGDR* levels increased in treated cells compared to the values obtained with ethanol (p-value = 0.007). Differences detected did not reach statistical significance, although at both times the wild-type CTCT showed a higher response than the mutated variants (Fig 4B).

### Promoter SNPs determined different putative transcription factors binding patterns

Different transcription factor binding patterns were obtained in the *in-silico* study using MatInspector and Transfac (BioBase) platforms. We observed the loss of a putative biding site for the glucocorticoid receptor (GR) in sequences carrying the mutant C variant at -549 polymorphic position. The C**C**CC, C**C**CT and T**C**CT had purportedly lost this GR binding site vs the wt CTCT. In addition, other putative binding differences between wild and mutated alleles at the -643, -549 and -197 positions were observed. These changes affected the binding affinity of GZF1, CAR/RXR, NBRE, PPARG, VDR/RXR, SOX15, MAF, GABP, FREAC2, NRSF, MAZ, SFR, ZFP652, NFAT, ISGF-3, and GABP transcription factors (Table 1).

**Table 1:** *In-silico* analysis of transcription factor binding site (TFBS) patterns obtained with MatInsprector software and Transfac (BioBase). The 653bp construct includes -613C>T, -549T>C, -441C>T and -197T>C polymorphic sites.

		-613 C>T	-549 T>C	-441 C>T	-197 T>C
**MatInspector**	Wild-type	E2F GZF1*	BACH1 **CAR/RXR** **PPARG** MEF2 HNF3B EVI1 **NBRE** PPARG **VDR/RXR** BCL6	LXRE **NRSF** ZNF263 THRB	E2F1 SP2 KKLF EOMES
	Mutated	E2F **SOX15** **MAFB** **MAFF**	BACH1 MEF2 HNF3B EVI1 PPARG **ZFP652** **PAX6** **GABP** BCL6 **FREAC2**	LXRE **PPAR/RXR** ZNF263 THRB	E2F1 SP2 **MAZ** **SRF** KKLF EOMES
**Transfac (Biobase)**	Wild-type		**NF-ATp** **ISGF-3** **NF-ATc,** **NF-ATp,** **NF-ATx** **SF-1** **GR**		
	Mutated				**IRE-ABP, SRY** **SP1** **LyF-1** **AP-2alphaA** **AP-2alphaB** **FREAC2** **CBF, SRF**

*Bold type transcription factors indicate differences between wild-type and mutated polymorphism.

### Approach to the cytokines secretion study

To explore the relationship between different levels of *PTGDR* and the development of the inflammatory cascade we analyzed the cytokine expression in cell cultures. For this, we used the culture media of different A549 cells transfected with expression vectors carrying the *PTGDR* gene under each of the four promoter haplotypes.

First, we observed that after 12 hours of culture, cells carrying expression vectors with the *PTGDR* promoter haplotypes CTCT (wt), CCCC and CCCT had higher cytokine levels than control cells (without expression vector). At this time, cells carrying the wild-type haplotype produced the highest concentrations for almost all cytokines measured (S1 Table). In contrast, after 36 hours of culture, cells carrying the wild-type CTCT sequence had lower values for most of tested cytokines than control cells and cells carrying mutated haplotypes.

When cells were treated with dexamethasone, we observed again a different responsiveness pattern depending on the promoter sequence present. After 12 hours of treatment, cells transfected with the wild-type CTCT variant, had decreased levels of cytokines, especially for IP-10, MCP-1, RANTES, MIP-1β, IL-6 and IL-8. After 36 hours of DEX treatment values of eotaxin and FGF-basic were also reduced in CTCT cells. However, in cells carrying any of the mutant haplotypes, the response degree based on almost all measures cytokines were less pronounced than the response degree observed in cells with wild-type CTCT sequence.

## Discussion

The present study focuses on the analysis of the influence of certain polymorphisms of the promoter region of the *PTGDR* gene on its promoter activity and gene expression, as well as in the evaluation of the effect of dexamethasone treatment. The study included a complete analysis at three different levels: promoter activity, gene expression and cytokine secretion. In this study, transfection of different haplotype combinations of *PTGDR* caused different promoter activities in A549 cells.

The combination of SNPs at four positions influenced not only the promoter activity but also expression levels of *PTGDR* and cytokine secretion patterns, which reinforce the idea that different SNPs located at different positions should be considered as a whole. In this work, for the first time, we have studied the promoter activity of the *PTGDR* gene in cell cultures, analysing the effect of the presence of gene variants in the four more frequently described positions, i.e. -613, -549, -441 and -197 [31]. Previous studies on the influence of *PTGDR* promoter polymorphisms did not included polymorphisms at -613C>T [19]. In this study, the simultaneous presence -613T and -549C in a haplotypic combination (TCCT) caused a significant decrease of the promoter activity after 12 and 36 hours of culture in basal conditions. This variant also showed lower levels in expression assays, an independent experimental approximation, suggesting that in the presence of both mutations, -613T and -549C (TCCT), a lower promoter activity would occur, leading to a lower expression of *PTGDR*.

On the other hand, the experimental data showed that the CCCC construction, which combines both -549C and -197C mutated alleles, caused a significant increase in the promoter activity at 12 and 36 hours (basal conditions). This increased promoter activity reinforces previous results showing that the CCC (-549, -441, -197) combination was associated with higher luciferase expression at 12 and 48 hours [32]. In addition, in our expression experiment, both CCCC and CCCT haplotypes showed higher expression levels that the CTCT wild-type *PTGDR* haplotype (-613, -549, -441 and -197 positions). Our results are consistent with our own studies, linking high levels of *PTGDR* mRNA expression in peripheral blood of patients with asthma (San Segundo Val et al., manuscript in preparation), and studies linking the -549T>C and -197T>C polymorphism with allergic asthma [19,20]. In addition, we have described that the diplotype CCCC CCCT was the most frequent diplotype in patients with asthma [21]. Therefore, the -549C SNP, individually (CCCT), or in combination with the -197T SNP (CCCC) would be associated to higher levels of expression than the wild-type *PTGDR* haplotype. Nevertheless, gene variants in the promoter region of *PTGDR* could lead to differences in expression regulation of this gene, which could be associated with an increased likelihood of developing allergic diseases. The correlation of in vitro expression and dyplotypic combinations in patients must be considered with caution, since other factors could influence the *PTGDR* expression and allergy development.

Several polymorphisms have been involved in corticosteroid responsiveness in asthma [33–39]. In this study we analyzed for the first time the effect of the treatment with corticosteroids taking into account different haplotypic combinations of the *PTGDR* promoter region. The addition of dexamethasone to the culture caused a different level of response, depending on the *PTGDR* haplotype. In the luciferase assays, corticosteroids induced an increment of the promoter activity both at 12h and 36h. Interestingly, the expression assay exhibited a two-phase response, with decreased levels of *PTGDR* at 12h, and increased levels at 36 hours, which could be indicating a time dependent biphasic mechanism of action of corticosteroids.

In all cases, cells carrying the wild-type variant CTCT had the greatest response, surpassing even the CCCC haplotype, i.e., the variant with the higher basal activity. It is noteworthy that these results were obtained in both promoter activity assays and expression analyses. On the other hand, we observed that the TCCT haplotype, which had the lowest values in basal conditions, also had the lowest response to dexamethasone treatment. The simultaneous presence of -613T and -549C (TCCT) could indicate that patients with these mutations located in the promoter region of *PTGDR* may respond less to treatment. Further studies are needed to confirm the role of this haplotype at the pharmacogenetic response.

Our data also suggest that the combination carrying non mutated SNPs at the four positions -613, -549, -441 and -197, had a higher capacity to respond to corticoid treatment, suggesting the presence of specific response elements in the wild-type variant, which are lost with polymorphic changes. In fact, our bioinformatics analysis showed that a differential allelic occupancy at these four positions determined modifications in the transcription factor binding, which could mediate differential performances. The presence of a thymine at -549 position, which only occurs in the wild-type haplotype, provides a potential binding site for the glucocorticoid receptor, which is lost in case of mutation. This could contribute to the worst response to dexamethasone observed in the CCCC, CCCT and TCCT variants, with respect to the wild CTCT variant (-613, -549, -441 and -197 positions). Moreover, only the wild-type variant bound to CAR/RXR and VDR/RXR heterodimers and GABP and FREAC transcription factors, involved in modulating the inflammatory state, balances between Th1/Th2 responses, and corticoid responses [40–47].

In the other hand, the -613C>T change, present only in the TCCT haplotype, caused the appearance of putative binding sites for SOX15 and for MAFB, which have been associated to pulmonary diseases [48,49]. Moreover, this mutation caused the loss of binding sites for GZF1, also called ZNF336. This transcription factor has a glucocorticoid response element [50], which could explain the different response to dexamethasone observed in the TCCT variant.

To further investigate the involvement of *PTGDR* action in inflammation, a multiplexed analysis of a wide range of cytokines was performed. Cell cultures with high *PTGDR* levels showed greater cytokine expression levels than control cells. This data suggest a putative role of *PTGDR* in the inflammatory response. Thus, cells with mutant haplotypes in the *PTGDR* promoter (CCCC, CCCT and TCCT) showed a higher increment in their cytokine levels than control cells in both experimental times analyzed. However, cells with wild-type variant CTCT produced lower cytokine levels than control cells when experimental time was prolonged until 36 hours. Data showed a restraint on increasing cytokines in cells expressing the wild variant of *PTGDR* with respect to other variants. It seems that the presence of the wild-type combination in the *PTGDR* promoter influences the evolution of cytokine levels. This could contribute to a lower predisposition to the allergic process. In addition, the presence of different *PTGDR* promoter haplotypes appears to cause different response after dexamethasone treatment. These preliminary data suggest that cells harbouring the wild-type haplotype have a better response to glucocorticoid showing a more pronounced decrease of proinflammatory cytokines, including cytokines and chemokines associated with asthma and Th2 lung inflammation, as IP-10 and RANTES [51,52], MIP-1β[53], IL-6, IL-8 [54], and eotaxin [55,56].

It appears that conserved nucleotides for -613C> T, -549T> C, -441C> T and -197T> C polymorphisms in the promoter *PTGDR*, as it happens in the wild haplotype CTCT, provide a greater overall response to treatment with dexamethasone, not only at gene expression level but also at cytokine expression level. As noted above, this variant is seen most frequently in control individuals [19,20,22] and seems also to be related to a greater sensitivity to corticosteroids.

In summary, we have shown the influence of *PTGDR* polymorphisms in the promoter activity and gene expression as well as in the secretory cytokine pattern in response to corticoid treatment. Therefore, it is important to note that these results could contribute to explain the variability in the corticoid response observed in the clinic. Deeper insight in the probable pharmacogenetic role of *PTGDR* in corticoid response is needed, in order to identify corticoid resistant allergic patients.

## Supporting information

S1 Table Influence of *PTGDR* variants on its promoter activity and response to corticoid treatment. (DOCX)

S2 Table Influence of *PTGDR* promoter variants on its expression levels and response to corticoid treatment. (DOCX)

S3 Table Influence of *PTGDR* promoter variants on the concentration of secreted cytokines. (DOCX)
