# Supplementary material for: PTGDR gene expression and response to dexamethasone treatment in an in vitro model
Source: PLoS One. 2017 Oct 31;12(10):e0186957. doi: 10.1371/journal.pone.0186957 (PMC5663384; doi:10.1371/journal.pone.0186957)
Supplement: S1 Table — (DOCX) [file pone.0186957.s001.docx]

**S1 Table. Influence of *PTGDR* variants on its promoter activity and response to corticoid treatment.**

| **No treatment** | |  |  |  |  |  |
| --- | --- | --- | --- | --- | --- | --- |
|  | **Haplotype** | **EXP1** | **EXP2** | **EXP3** | **MEAN** | **SD** |
| **12h** | CTCT | 0,09 | 0,10 | 0,11 | 0,10 | 0,01 |
|  | CCCC | 0,10 | 0,12 | 0,12 | 0,11 | 0,01 |
|  | CCCT | 0,09 | 0,11 | 0,11 | 0,10 | 0,01 |
|  | TCCT | 0,08 | 0,10 | 0,09 | 0,09 | 0,01 |
| **36H** | CTCT | 0,10 | 0,10 | 0,11 | 0,10 | 0,01 |
|  | CCCC | 0,10 | 0,10 | 0,11 | 0,10 | 0,00 |
|  | CCCT | 0,09 | 0,09 | 0,09 | 0,09 | 0,00 |
|  | TCCT | 0,07 | 0,07 | 0,08 | 0,08 | 0,00 |
| **No treatment normalized data** | | |  |  |  |  |
|  | **Haplotype** | **EXP1** | **EXP2** | **EXP3** | **MEAN** | **SD** |
| **12h** | CTCT | 1 | 1 | 1 | 1 | 0 |
|  | CCCC | 1,14 | 1,17 | 1,11 | 1,14 | 0,03 |
|  | CCCT | 1,01 | 1,04 | 1,07 | 1,04 | 0,03 |
|  | TCCT | 0,86 | 0,94 | 0,88 | 0,89 | 0,04 |
| **36H** | CTCT | 1,00 | 1,00 | 1,00 | 1,00 | 0,00 |
|  | CCCC | 1,06 | 1,02 | 1,01 | 1,03 | 0,03 |
|  | CCCT | 0,91 | 0,89 | 0,88 | 0,89 | 0,01 |
|  | TCCT | 0,76 | 0,74 | 0,75 | 0,75 | 0,01 |
| **Treatment** | |  |  |  |  |  |
|  | **Condition/Haplotype** | **EXP1** | **EXP2** | **EXP3** | **MEAN** | **SD** |
| **12 h** | EtOH CTCT | 0,10 | 0,11 | 0,11 | 0,11 | 0,01 |
|  | EtOH CCCC | 0,11 | 0,12 | 0,11 | 0,11 | 0,01 |
|  | EtOH CCCT | 0,09 | 0,11 | 0,11 | 0,11 | 0,01 |
|  | EtOH TCCT | 0,08 | 0,10 | 0,09 | 0,09 | 0,01 |
|  | DEX CTCT | 0,57 | 0,62 | 0,74 | 0,64 | 0,09 |
|  | DEX CCCC | 0,63 | 0,65 | 0,69 | 0,66 | 0,03 |
|  | DEX CCCT | 0,54 | 0,56 | 0,65 | 0,59 | 0,06 |
|  | DEX TCCT | 0,44 | 0,50 | 0,52 | 0,49 | 0,04 |
| **36h** | EtOH CTCT | 0,11 | 0,09 | 0,11 | 0,11 | 0,01 |
|  | EtOH CCCC | 0,11 | 0,10 | 0,10 | 0,10 | 0,01 |
|  | EtOH CCCT | 0,09 | 0,09 | 0,09 | 0,09 | 0,00 |
|  | EtOH TCCT | 0,08 | 0,07 | 0,08 | 0,08 | 0,00 |
|  | DEX CTCT | 0,37 | 0,31 | 0,44 | 0,37 | 0,06 |
|  | DEX CCCC | 0,36 | 0,27 | 0,41 | 0,35 | 0,07 |
|  | DEX CCCT | 0,27 | 0,24 | 0,35 | 0,29 | 0,06 |
|  | DEX TCCT | 0,25 | 0,21 | 0,31 | 0,25 | 0,05 |
| **Treatment Normalized data** | |  |  |  |  |  |
|  | **Haplotype** | **EXP1** | **EXP2** | **EXP3** | **MEAN** | **SD** |
| **12h** | CTCT | 1 | 1 | 1 | 1 | 0 |
|  | CCCC | 1,11 | 1,05 | 0,93 | 1,03 | 0,09 |
|  | CCCT | 0,96 | 0,89 | 0,87 | 0,91 | 0,05 |
|  | TCCT | 0,77 | 0,80 | 0,68 | 0,75 | 0,06 |
| **36H** | CTCT | 1 | 1 | 1 | 1 | 0 |
|  | CCCC | 0,988 | 0,788 | 0,956 | 0,911 | 0,107 |
|  | CCCT | 0,716 | 0,694 | 0,795 | 0,735 | 0,053 |
|  | TCCT | 0,666 | 0,614 | 0,705 | 0,661 | 0,046 |
