# Supplementary material for: PTGDR gene expression and response to dexamethasone treatment in an in vitro model
Source: PLoS One. 2017 Oct 31;12(10):e0186957. doi: 10.1371/journal.pone.0186957 (PMC5663384; doi:10.1371/journal.pone.0186957)
Supplement: S2 Table — (DOCX) [file pone.0186957.s002.docx]

S2 Table. Influence of *PTGDR* promoter variants on its expression levels and response to corticoid treatment.

| **No treatment** | |  |  |  |  |
| --- | --- | --- | --- | --- | --- |
|  | **Haplotype** | **EXP1** | **EXP2** | **Mean** | **SD** |
| **12h** | CTCT | 1476,88 | 6936,54 | 4206,71 | 3860,56 |
|  | CCCC | 1490,59 | 7332,05 | 4411,32 | 4130,53 |
|  | CCCT | 1449,83 | 8306,36 | 4878,09 | 4848,3 |
|  | TCCT | 1324,9 | 3623,91 | 2474,41 | 1625,65 |
| **36H** | CTCT | 10846,94 | 8769,83 | 9808,38 | 1468,74 |
|  | CCCC | 11679,3 | 10285,56 | 10982,43 | 985,52 |
|  | CCCT | 8609,22 | 11255,43 | 9932,32 | 1871,15 |
|  | TCCT | 9821,08 | 8589,35 | 9205,22 | 870,97 |
| **No treatment normalized data** | | |  |  |  |
|  | **Haplotype** | **EXP1** | **EXP2** | **Mean** | **SD** |
| **12h** | CTCT | 1 | 1 | 1 | 0 |
|  | CCCC | 1,01 | 1,06 | 1,03 | 0,03 |
|  | CCCT | 0,98 | 1,20 | 1,09 | 0,15 |
|  | TCCT | 0,90 | 0,52 | 0,71 | 0,27 |
| **36H** | CTCT | 1 | 1 | 1 | 0 |
|  | CCCC | 1,08 | 1,17 | 1,12 | 0,07 |
|  | CCCT | 0,79 | 1,28 | 1,04 | 0,35 |
|  | TCCT | 0,91 | 0,98 | 0,94 | 0,05 |
| **Treatment** | |  |  |  |  |
|  | **Condition/Haplotype** | **EXP1** | **EXP2** | **Mean** | **SD** |
| **12h** | EtOH CTCT | 6653,97 | 21003,37 | 13828,67 | 10146,56 |
|  | EtOH CCCC | 6968,67 | 16747,60 | 11858,13 | 6914,75 |
|  | EtOH CCCT | 6872,73 | 19461,47 | 13167,10 | 8901,59 |
|  | EtOH TCCT | 7408,68 | 14230,18 | 10819,43 | 4823,53 |
|  | DEX CTCT | 2619,36 | 12677,65 | 7648,51 | 7112,29 |
|  | DEX CCCC | 2312,12 | 13937,33 | 8124,73 | 8220,27 |
|  | DEX CCCT | 2730,60 | 14099,27 | 8414,94 | 8038,87 |
|  | DEX TCCT | 2355,25 | 11883,45 | 7119,35 | 6737,46 |
| **36h** | EtOH CTCT | 23170,48 | 43038,30 | 33104,39 | 14048,67 |
|  | EtOH CCCC | 27939,14 | 46879,41 | 37409,27 | 13392,79 |
|  | EtOH CCCT | 25064,07 | 46879,41 | 35971,74 | 15425,77 |
|  | EtOH TCCT | 18305,63 | 42988,60 | 30647,12 | 17453,50 |
|  | DEX CTCT | 54350,29 | 98193,05 | 76271,67 | 31001,52 |
|  | DEX CCCC | 55492,30 | 100024,90 | 77758,61 | 31489,32 |
|  | DEX CCCT | 46234,00 | 102599,70 | 74416,84 | 39856,55 |
|  | DEX TCCT | 44865,95 | 95067,64 | 69966,79 | 35497,95 |
| **Treatment Normalized data** | |  |  |  |  |
|  | **Haplotype** | **EXP1** | **EXP2** | **Mean** | **SD** |
| **12h** | CTCT | 1 | 1 | 1 | 0 |
|  | CCCC | 1,15 | 0,34 | 0,75 | 0,58 |
|  | CCCT | 1,03 | 0,64 | 0,84 | 0,27 |
|  | TCCT | 1,25 | 0,28 | 0,77 | 0,68 |
| **36H** | CTCT | 1 | 1 | 1 | 0 |
|  | CCCC | 0,88 | 0,96 | 0,92 | 0,06 |
|  | CCCT | 0,68 | 1,01 | 0,84 | 0,23 |
|  | TCCT | 0,85 | 0,94 | 0,89 | 0,06 |
