# Supplementary material for: PTGDR gene expression and response to dexamethasone treatment in an in vitro model
Source: PLoS One. 2017 Oct 31;12(10):e0186957. doi: 10.1371/journal.pone.0186957 (PMC5663384; doi:10.1371/journal.pone.0186957)
Supplement: S3 Table — (DOCX) [file pone.0186957.s003.docx]

**S3 Table. Influence of *PTGDR* promoter variants on the concentration of secreted cytokines.**

| **Non-normalized data** | | | | | | | | | | | | | | | | | | | |
| --- | --- | --- | --- | --- | --- | --- | --- | --- | --- | --- | --- | --- | --- | --- | --- | --- | --- | --- | --- |
| **12 h (pg/ml)** | | **CONTROL** | | | **CTCT** | | | | **CCCC** | | | | **CCCT** | | | | **TCCT** | | |
|  |  | **POINT 1** | **POINT 2** | | **POINT 1** | | **POINT 2** | | **POINT 1** | | **POINT 2** | | **POINT 1** | | **POINT 2** | | **POINT 1** | **POINT 2** | |
| **IP-10** | | 2130,46 | 1908,99 | | 3158,91 | | 2911,52 | | 3105,74 | | 2631,53 | | 2467,03 | | 2472,05 | | 1444,46 | 3096,06 | |
| **MCP-1** | | 1578,63 | 1671,13 | | 1890,19 | | 1758,35 | | 1841,86 | | 1785,2 | | 1794,25 | | 1890,19 | | 1165,71 | 1823,26 | |
| **IL8** | | 659,3 | 638,33 | | 855,11 | | 816,04 | | 782,11 | | 746,13 | | 651 | | 778,23 | | 604,54 | 836,23 | |
| **RANTES** | | 484,76 | 430,05 | | 674,27 | | 607,15 | | 593,7 | | 519,82 | | 464,94 | | 485,69 | | 320,38 | 587,14 | |
| **VEGF** | | 353,58 | 350,99 | | 464,23 | | 443,87 | | 404,45 | | 379,98 | | 398,95 | | 393,49 | | 148,25 | 423,95 | |
| **MIP-1B** | | 53,22 | 50,45 | | 85,47 | | 84,47 | | 76,6 | | 73,59 | | 63,07 | | 67,65 | | 43,36 | 81,61 | |
| **IL12** | | 44,77 | 47,45 | | 70,17 | | 60,44 | | 58,54 | | 54,78 | | 50,17 | | 48,35 | | 23,26 | 62,36 | |
| **IL6** | | 24,55 | 23,6 | | 40,24 | | 37,93 | | 40,45 | | 35,13 | | 31,19 | | 32,79 | | 25,12 | 36,37 | |
| **IL7** | | 23,18 | 28,78 | | 28,78 | | 31,63 | | 31,63 | | 25,96 | | 25,96 | | 25,96 | | 20,45 | 28,78 | |
| **36h (pg/ml)** | | **CONTROL** | | | **CTCT** | | | | **CCCC** | | | | **CCCT** | | | | **TCCT** | | |
|  |  | **POINT 1** | **POINT 2** | | **POINT 1** | | **POINT 2** | | **POINT 1** | | **POINT 2** | | **POINT 1** | | **POINT 2** | | **POINT 1** | **POINT 2** | |
| **IL8** | | 1672,46 | 1569,49 | | 1580,14 | | 1431,58 | | 2230,24 | | 2158,69 | | 2546,55 | | 1238,67 | | 1667,56 | 1928,54 | |
| **MCP1** | | 1621,22 | 800,73 | | 1408,18 | | 683,3 | | 1553,39 | | 1296,34 | | 1635,64 | | 1244 | | 1357,02 | 1310,49 | |
| **VEGF** | | 935,43 | 963,59 | | 671,37 | | 770,99 | | 1097,92 | | 1207,87 | | 1774,86 | | 333,05 | | 592,4 | 1523,81 | |
| **INF-g** | | 631,37 | 330,63 | | 436,73 | | 330,63 | | 590,79 | | 590,79 | | 672,72 | | 233,74 | | 418,45 | 631,37 | |
| **MIP-1B** | | 171,84 | 148,42 | | 148,93 | | 144,97 | | 239,92 | | 285,15 | | 306,62 | | 87,58 | | 105,17 | 265,57 | |
| **IL6** | | 85,67 | 85,02 | | 78,71 | | 107,39 | | 134,07 | | 146,46 | | 166,48 | | 63,07 | | 83,72 | 131,94 | |
| **IL12** | | 113,44 | 114,56 | | 85,39 | | 77,18 | | 143,4 | | 145,78 | | 175,12 | | 47 | | 70,66 | 155,4 | |
| **EOTAXINA** | | 73,55 | 51,03 | | 55,94 | | 55,94 | | 77,61 | | 73,55 | | 81,58 | | 45,77 | | 60,61 | 81,58 | |
| **B-FGF** | | 56,32 | 45,27 | | 54,5 | | 44,75 | | 74,57 | | 67,42 | | 87,28 | | 40,99 | | 66,6 | 79,16 | |
| **IL7** | | 52,54 | 46,4 | | 37,44 | | 40,39 | | 55,66 | | 52,54 | | 58,8 | | 23,18 | | 31,63 | 65,18 | |
| **IL1ra** | | 63,72 | 38,5 | | 42,67 | | 34,35 | | 72,25 | | 63,72 | | 80,85 | | 30,22 | | 51,04 | 67,98 | |
| **IL5** | | 23,79 | 15,84 | | 17,6 | | 15,84 | | 27,33 | | 28,21 | | 28,21 | | 14,07 | | 14,95 | 25,56 | |
| **TNFa** | | 22,1 | 13,36 | | 14,46 | | 11,18 | | 23,2 | | 23,2 | | 24,29 | | 11,18 | | 15,55 | 23,2 | |
| **Normalized data** | | | | | | | | | | | | | | | | | | | |
| **12 h** | **CONTROL** | | | **CTCT** | | | | **CCCC** | | | | **CCCT** | | | | **TCCT** | | | |
|  |  |  |  | **MEAN** | | **SD** | | **MEAN** | | **SD** | | **MEAN** | | **SD** | | **MEAN** | | | **SD** |
| **IP-10** | 1 | | | 1,50 | | 0,09 | | 1,42 | | 0,17 | | 1,22 | | 0,00 | | 1,12 | | | 0,58 |
| **MCP-1** | 1 | | | 1,12 | | 0,06 | | 1,12 | | 0,02 | | 1,13 | | 0,04 | | 0,92 | | | 0,29 |
| **IL8** | 1 | | | 1,29 | | 0,04 | | 1,18 | | 0,04 | | 1,10 | | 0,14 | | 1,11 | | | 0,25 |
| **RANTES** | 1 | | | 1,40 | | 0,10 | | 1,22 | | 0,11 | | 1,04 | | 0,03 | | 0,99 | | | 0,41 |
| **VEGF** | 1 | | | 1,29 | | 0,04 | | 1,11 | | 0,05 | | 1,12 | | 0,01 | | 0,81 | | | 0,55 |
| **MIP-1β** | 1 | | | 1,64 | | 0,01 | | 1,45 | | 0,04 | | 1,26 | | 0,06 | | 1,21 | | | 0,52 |
| **IL12** | 1 | | | 1,42 | | 0,15 | | 1,23 | | 0,06 | | 1,07 | | 0,03 | | 0,93 | | | 0,60 |
| **IL6** | 1 | | | 1,62 | | 0,07 | | 1,57 | | 0,16 | | 1,33 | | 0,05 | | 1,28 | | | 0,33 |
| **IL7** | 1 | | | 1,16 | | 0,08 | | 1,11 | | 0,15 | | 1,00 | | 0,00 | | 0,95 | | | 0,23 |
| **36 h** | **CONTROL** | | | **CTCT** | | | | **CCCC** | | | | **CCCT** | | | | **TCCT** | | | |
|  |  |  |  | **MEAN** | | **SD** | | **MEAN** | | **SD** | | **MEAN** | | **SD** | | **MEAN** | | | **SD** |
| **IL8** | 1 | | | 0,93 | | 0,06 | | 1,35 | | 0,03 | | 1,17 | | 0,57 | | 1,11 | | | 0,11 |
| **MCP1** | 1 | | | 0,86 | | 0,42 | | 1,18 | | 0,15 | | 1,19 | | 0,23 | | 1,10 | | | 0,03 |
| **VEGF** | 1 | | | 0,76 | | 0,07 | | 1,21 | | 0,08 | | 1,11 | | 1,07 | | 1,11 | | | 0,69 |
| **INF-γ** | 1 | | | 0,80 | | 0,16 | | 1,23 | | 0,00 | | 0,94 | | 0,65 | | 1,09 | | | 0,31 |
| **MIP-1β** | 1 | | | 0,92 | | 0,02 | | 1,64 | | 0,20 | | 1,23 | | 0,97 | | 1,16 | | | 0,71 |
| **IL6** | 1 | | | 1,09 | | 0,24 | | 1,64 | | 0,10 | | 1,34 | | 0,86 | | 1,26 | | | 0,40 |
| **IL12** | 1 | | | 0,71 | | 0,05 | | 1,27 | | 0,01 | | 0,97 | | 0,79 | | 0,99 | | | 0,53 |
| **EOTAXIN** | 1 | | | 0,90 | | 0,00 | | 1,21 | | 0,05 | | 1,02 | | 0,41 | | 1,14 | | | 0,24 |
| **FGF basic** | 1 | | | 0,98 | | 0,14 | | 1,40 | | 0,10 | | 1,26 | | 0,64 | | 1,43 | | | 0,17 |
| **IL7** | 1 | | | 0,79 | | 0,04 | | 1,09 | | 0,04 | | 0,83 | | 0,51 | | 0,98 | | | 0,48 |
| **IL1ra** | 1 | | | 0,75 | | 0,12 | | 1,33 | | 0,12 | | 1,09 | | 0,70 | | 1,16 | | | 0,23 |
| **IL5** | 1 | | | 0,84 | | 0,06 | | 1,40 | | 0,03 | | 1,07 | | 0,50 | | 1,02 | | | 0,38 |
| **TNFα** | 1 | | | 0,72 | | 0,13 | | 1,31 | | 0,00 | | 1,00 | | 0,52 | | 1,09 | | | 0,31 |
